# Management of child maltreatment suspicions in general practice: a mixed methods study

**DOI:** 10.1080/02813432.2023.2178851

**Published:** 2023-02-22

**Authors:** Camilla Hoffmann Merrild, Hans Christian Kjeldsen, Ioanna Milidou

**Affiliations:** a Center for General Practice, Aalborg University, Aalborg Ø, Denmark; b Lægefællesskabet Grenå, Aarhus University, Denmark; c Department of Child and Adolescent Medicine, Gødstrup Regional Hospital

**Keywords:** Primary care, child health, maltreatment, abuse, diagnostics

## Abstract

**Background:**

Maltreated children have many long-term consequences throughout their lives, but often maltreated children are not recognised in time by professionals. General practice could be central to the early recognition of child maltreatment due to the long-term relationship with families.

**Objective:**

How do general practitioners (GPs) and practice nurses (PNs) deal with suspected maltreatment in children below 18 years of age, and which factors influence them to report cases to social authorities.

**Design and setting:**

A mixed methods study set in general practice in Denmark.

**Method and subjects:**

We combined data from a nationwide questionnaire with observations from five clinics and 20 interviews with GPs and PNs. We explored our data using the concept of uncertainty as a driver that shapes action and decision-making in general practice.

**Results:**

Most GPs (94%) said they would discuss cases of suspected child maltreatment with social services, but in many cases they would prefer to discuss their suspicions with a colleague first (83%) – most likely where there are no clear-cut signs. The qualitative data added nuance to these findings by highlighting the difficulty of communicating across sectors, the importance of maintaining a connection with the child’s family, and practicing watchful waiting.

**Conclusion:**

General practice has an opportunity to act early in cases of suspected child maltreatment if uncertainty is accepted as a critical part of the process of reaching a diagnosis. Communication across sectors is key, as is support for GPs with suspicions and for families in need of help.

**Key points**
- GPs are often thought to underreport child maltreatment but despite low levels of reporting, this does not mean they ignore it.
- Building on the connection with the family, making follow appointments, and discussing suspicions with colleagues are typical of how GPs manage suspicions of child abuse.
- Accepting uncertainty as a condition of raising the alarm could help GPs to act quickly to support children at risk of abuse.

## Introduction

Child maltreatment is a major public-health and social-welfare problem, with dramatic consequences for the victim’s physical, mental, and emotional health throughout childhood and adult life [1]. WHO [2] defines child maltreatment as the abuse and neglect that occurs to children under 18 years of age, including all types of physical and/or emotional ill-treatment, sexual abuse, neglect, negligence and commercial or other exploitation, which results in actual or potential harm to the child’s health, survival, development or dignity in the context of a relationship of responsibility, trust or power. A recent meta-analysis shows significantly increased health-related and economic costs resulting from adverse childhood experiences across all European countries [3]. Reports to the social authorities in cases of suspicion of abuse and neglect are mandatory for all citizens in Denmark. However, those who work professionally with children, including health care professionals in all settings, workers in schools, kindergartens, daycare etc., and workers in the sectors of care and support of people with social or other special needs and challenges, have an extended obligation to react when there is a presumption that a child needs help. It has been suggested, however, that up to 90% of child maltreatment goes unnoticed [4]. Studies with adult victims of childhood abuse and neglect describe how victims felt overlooked or ignored by health professionals, even though they considered their precarious situation to be obvious to outsiders [5,6]. Likewise, it has been shown that children of substance abusers or patients with mental illness often lacked recognition of their precarious situation by their GP [7].

General practice is the front line of the health care system in Denmark and provides expense-free health care visits on demand. Approximately 20% of regular consultations in Danish general practice are with children, and cover everything from three scheduled prophylactic child-well visits during the first year and annual visits until the child turns five combined with immunisations and ad hoc contacts, often with infections or injuries. More than 90% of children attend the first three child-well visits, after which attendance seems to decline slightly [8]. Thus, the general practitioner (GP) and sometimes the practice nurse (PN) may be the most consistent health professional in children’s lives, as they follow them from pregnancy throughout their childhood. Continuity of care is a core principle of the way that general practice is organised, as is timely diagnosis and prioritising those whose needs are greatest [9]. This positions general practice as central in early recognition and reporting of child maltreatment. The longitudinal contact between the GP, PN, the child, and the rest of the family may offer opportunities to identify children at risk. It has been argued that GPs seem reluctant to report on their suspicions of child maltreatment [1,10], possibly due to a lack of knowledge about symptoms and how to deal with suspicions, uncertainty about the diagnosis, and fear of impeding the relationship with the family [11–13]. In a pilot study we found that in cases of obvious signs of maltreatment, GPs are not in any doubt about how to proceed [14]. However, in the complex reality of clinical general practice, GPs are faced with a wide range of different child health concerns, which rarely offer room for suspicion when signs are unclear [14–16]. Moreover, a Norwegian study of children as next of kin to parents with mental illness or substance abuse, have shown that although GPs may have an important supportive role to play for ‘invisible’ children, they often miss the opportunity to do so, due to working conditions in general practice [17]. Little is known about how suspicions of child maltreatment are managed in a general practice context. In this article, we seek to direct attention towards what happens in that space before reports to social services are made, or not made. In order to address this knowledge gab, we explore the question how Danish GPs and PNs deal with suspicions of child maltreatment, what actions they take, and which challenges they face.

## Methods

Our study was designed as a convergent, parallel, mixed methods approach [18], combining observations of consultations and interviews with GPs and PNs, and questionnaires with GPs. In order to understand different aspects of how suspicions of child maltreatment are managed in clinical practice, we wanted to combine quantitative and qualitative data to generate a more complete and detailed understanding of the topic under investigation. We combined a nationwide questionnaire completed by GPs and ethnographic fieldwork, consisting of interviews with GPs and PNs with observations in different general practice clinics in the period October 2019 through June 2020. Data collection in the two studies was carried out simultaneously, and meetings were held continuously throughout the study period, to discuss progress and provisional findings as they emerged.

### Questionnaire

#### Data collection

In October 2019 we sent a questionnaire to all registered doctors working in GP in Denmark, exploring doctors’ knowledge, experience, attitudes, and personal involvement with child abuse and neglect. The respondents are presented in Table 1. We used a validated Danish translation of a questionnaire originally developed for dentists and dental hygienists [19]. Data collection was completed in June 2020.

**Table 1.** Participants characteristics, background population, attitudes and preferences about reporting child abuse or neglect among Danish General Practitioners.

	Participants, n (%)	Danish GPs,^a^ N (%)	If you suspect child abuse/ neglect, will *rather* notify, or discuss with^b, c^n (%)				Would rather discuss *before* notification with^c^n (%)			
			Social services	Police	Colleagues	Caregiver	Colleagues	School, daycare, kindergarten	Child’s family, caregiver	Worker in the social services
Sex										
Male	512 (41)	1,441 (43)	482 (94)	59 (12)	260 (51)	271 (53)	410 (80)	11 (2)	25 (5)	32 (6)
Female	711 (57)	1,885 (57)	670 (94)	65 (9)	507 (71)	467 (66)	608 (86)	27 (4)	61 (9)	75 (11)
Age										
31–40	110 (9)	231 (7)	108 (98)	15 (14)	79 (72)	68 (62)	99 (90)	1 (1)	4 (4)	11 (10)
41–50	496 (40)	1,366 (40)	473 (95)	59 (12)	337 (68)	320 (65)	427 (86)	14 (3)	35 (7)	47 (10)
51–60	362 (29)	972 (29)	338 (93)	30 (8)	237 (66)	209 (58)	299 (83)	14 (4)	29 (8)	36 (10)
60+	258 (21)	757 (23)	236 (92)	20 (8)	115 (45)	139 (54)	196 (76)	9 (4)	4 (4)	13 (5)
Type of general practice										
Group practice	922 (74)	2,617 (79)	860 (95)	88 (10)	636 (70)	559 (61)	808 (89)	27 (3)	57 (6)	77 (9)
Single practice	183 (15)	709 (21)	167 (92)	16 (9)	57 (31)	100 (55)	105 (58)	8 (4)	13 (7)	19 (10)
Collaboration practice	108 (9)		114 (96)	19 (16)	70 (59)	71 (60)	96 (81)	2 (2)	14 (12)	9 (8)
Town/city	676 (54)	No data	638 (94)	65 (10)	422 (62)	414 (61)	567 (84)	14 (2)	56 (8)	60 (9)
Country	189 (15)	No data	176 (93)	19 (10)	112 (59)	106 (56)	157 (83)	10 (5)	11 (6)	21 (11)
Mixed	360 (29)	No data	340 (94)	39 (11)	234 (52)	216 (60)	296 (82)	13 (4)	19 (15)	27 (8)
Region										
Capital region	343 (27)	1033 (31)	325 (95)	35 (10)	205 (60)	215 (63)	279 (81)	12 (4)	26 (8)	36 (11)
Zealand	139 (11)	435 (13)	129 (93)	17 (12)	82 (59)	86 (62)	114 (82)	8 (2)	12 (9)	10 (7)
Southern Denmark	286 (23)	775 (23)	263 (92)	32 (11)	182 (64)	172 (60)	238 (83)	10 (4)	23 (8)	28 (10)
Central Denmark	374 (30)	798 (24)	354 (95)	31 (8)	251 (67)	213 (57)	314 (84)	8 (2)	20 (5)	31 (8)
North Denmark	105 (8)	285 (9)	103 (98)	13 (12)	59 (56)	60 (57)	87 (83)	2 (2)	8 (8)	4 (4)
Total	1252		1179 (94)	128 (10)	782 (63)	751 (60)	1041 (83)	39 (3)	90 (7)	110 (9)

*Note:* Percentages are rounded to the nearest integer. Percentage of missing values: Region: 0.4%; sex, age, type of GP practice: 1.9-2.3%.

^a^According to the Danish GPs’ Association report (2020).

^b^More than one replies were possible.

^c^120 responders used the free-text option: hospital colleagues (4.8%); school/kindergarten/daycare (1.2%); health visitor (0.8%); own network (0.6%); social services (0.6%); others from the child’s network (0.2%), and lawyer from the medical association (0.01%). Answers did not differ according to sex, age, type of practice, or Region.

#### The questionnaire

In the present study we present the part of the questionnaire concerning management of suspicions of child maltreatment among GP doctors.

Two questions explored the preferences of GPs in cases where they suspect child maltreatment. The first addressed concrete suspicions: who will the GP prefer to report to, or discuss with, if he/she suspects child abuse or neglect. More than one replies were possible among the four suggested (social services, police, colleague(s), caregiver, y/n), as well as free-text. The second question had two arms, and explored whether the GP would prefer to discuss the case with a colleague before reporting to the social authorities (y/n), and whether he/she would prefer to discuss with other professionals (y/n, free-text) *before* reacting to a suspicion in a hypothetical case of child abuse or neglect. Finally, the questionnaire included several factors (listed in Figure 2) and explored their possible influence (y/n) in the GPs’ decision to report to social services; more than one replies were possible. Three factors (fear of breaking the legislation, fear of doing something wrong, cooperation with the family) were added to the original questionnaire by the authors based on personal communications and experiences.

#### Statistical analysis

To explore possible demographic and geographic differences among GPs, we stratified the questionnaire responses according to sex, age, type of practice (group, single, collaboration between several practices; town/city, country, mixed), and geographical area (five Danish regions: Capital Region, Zealand, Central Region of Denmark, North Denmark Region, Southern Denmark Region).

### Fieldwork and interviews

#### Data collection

The qualitative data were based on five weeks of observation in five different general practice clinics, and 20 interviews with GPs and PNs, carried out by the first author between November 2019 and March 2020. Participating practices were strategically sampled [20], to ensure variation in practice type, geographical location, setting (rural, urban, provincial), and patient population (sociodemographic composition). The first author spent one week at each clinic with different doctors, nurses and patients, and observed hundreds of consultations covering a wide range of health-related problems, not only child consultations. This proved invaluable in developing a contextual understanding of how GPs and PNs think about and develop concerns, diagnoses, and care for patients. The first author also interviewed 20 GPs and PNs, all of whom had experience with child consultations. Some worked at the clinics where observations were carried out, and others were recruited from different clinics, locations, and patient populations through purposive sampling. Table 2 provides an overview of the GPs and PNs who were interviewed.

**Table 2.** Interview persons.

Profession	Years of experience in general practice	Age	Gender	Practice type
GP1	29	66	F	Single practice, rural
GP2	3	39	F	Collaboration practice, town
GP3	28	65	F	Single practice, rural
PN1	3	57	F	Collaboration practice, town
GP4	7	51	F	Collaboration practice, rural
GP5	1	39	F	Collaboration practice, rural
GP6	17	65	M	Single practice, urban
PN2	3	37	F	Collaboration practice, town
GP7	22	54	M	Collaboration practice, town
GP8	6	46	M	Collaboration practice, rural
GP 9	8	53	M	Collaboration practice, urban
GP10	14	53	F	Collaboration practice, rural
GP11	15	56	F	Collaboration practice, rural
GP12	10	46	F	Collaboration practice, urban
GP13	12	49	M	Collaboration practice, urban
PN 3	15	59	F	Single practice, rural
GP14	14	54	F	Collaboration practice, urban
GP15	1	32	F	Collaboration practice, urban
PN4	4	38	F	Collaboration practice, urban
PN5	3	36	F	Collaboration practice, urban

The choice to include both GPs and PNs was based on recent developments in Danish general practice, where more consultations are handled by practice nurses, such as child vaccinations and child well visits. The interviews focussed on experiences with reporting on child maltreatment, perceptions of what child maltreatment is and how it may manifest, and child welfare in the context of general practice.

#### Data analysis

**Figure 1.**
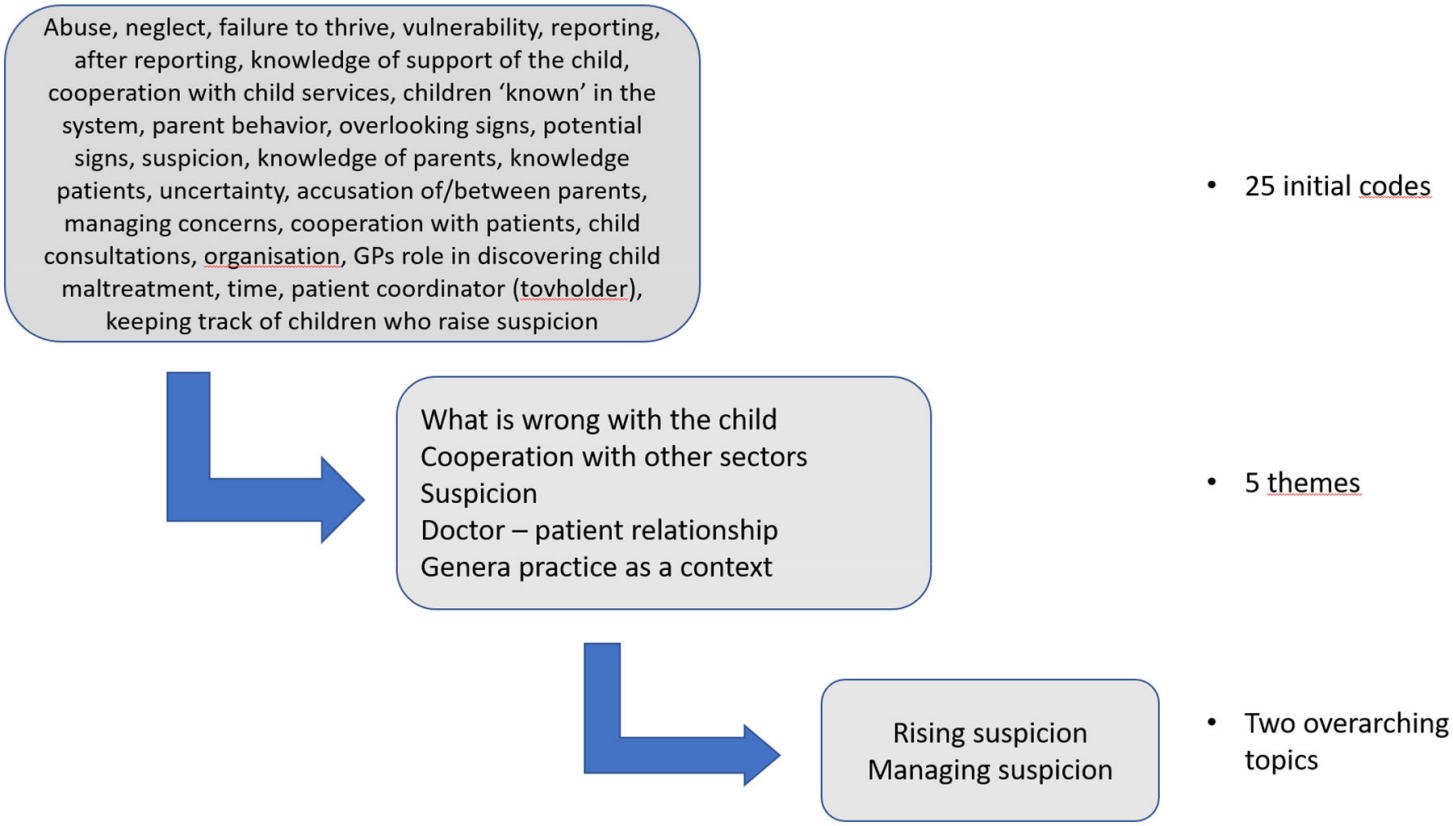
Coding process.

All interviews were transcribed verbatim and, together with field notes, read several times to develop an overview of patterns and overarching themes. Subsequently, the first author carried out an open coding using Nvivo13 and developed 25 codes, which were grouped into five themes: what is wrong with the child; cooperation with other sectors; suspicion; the doctor-patient relationship, and general practice as a context. Figure 1 illustrates the coding process.

The themes were then discussed within the research group, which was made up of two forensic specialists in child abuse, one GP, two paediatricians with experience in the field of maltreatment, and one anthropologist with research experience from general practice.

### Theoretical perspective

To make sense of how, when, and why suspicions of maltreatment arise in general practice, studies have applied theoretical concepts such as intuition [21] and gut feeling [15,22]. We explore this through the concept of uncertainty which is increasingly recognised as a condition for practicing medicine [23] and is intrinsic to making choices (on treatment, procedures, medication etc.). As noted by Professor of general practice Guri Rortveit; uncertainty ‘is a core concept of medical activity, especially in general practice, where illness is evaluated at an early stage and available diagnostic tools are limited’ [24,p.135]. Within social sciences, research focus on understanding how uncertainty is dealt with and made sense of in social situations [25], what it means to people living in particular situations and contexts, and how it is experienced and managed in daily life [26]. We try to bridge the medical and social approaches as we explore how the need for support amid feelings of uncertainty may be an important aspect of diagnosing child maltreatment in situations where there are no concrete biological signs or indications, but still ‘something’ which alerts the attention of the health professional.

## Results

Below we present the results from the quantitative and qualitative studies separately and subsequently we discuss them in combination.

### Questionnaire

#### Attitudes, preferences and factors affecting the decision to make a mandatory report

We sent 3,429 questionnaires to all GPs in Denmark and 1,252 completed questionnaires were returned (response rate 37.6%). 512 (41%) of the respondents were male, and 1,233 (98%) had finished specialty training in general practice. Data on the background total Danish GP population (*[Doctors and practice population 1997–2020 Key figures from the members registry]*, 2020) is shown for comparison (Table 1).

Among the options suggested by the questionnaire, the GPs preferred to report or discuss with social services (94%); a colleague (63%); the caregiver (60%); and the police (10%) in case of suspected child abuse and/or neglect. Generally, no large differences were observed across strata, except for the number of GPs who would notify or discuss a case with colleagues (mean 63%; range 31 -72%). This option was reported by less than one third of GPs working alone, and by more than two thirds of female and younger GPs.

Most GPs (83%), especially female (86%), younger (90%), and working in a group practice (88.8%), would rather discuss cases with (a) colleague(s) before making a report. A few GPs would discuss the issue with the child’s school, kindergarten, or daycare, the child’s caregiver, or family, or with someone from social services. In general, no large differences were observed across sex, age, type of practice, or region (Table 1).

**Figure 2.**
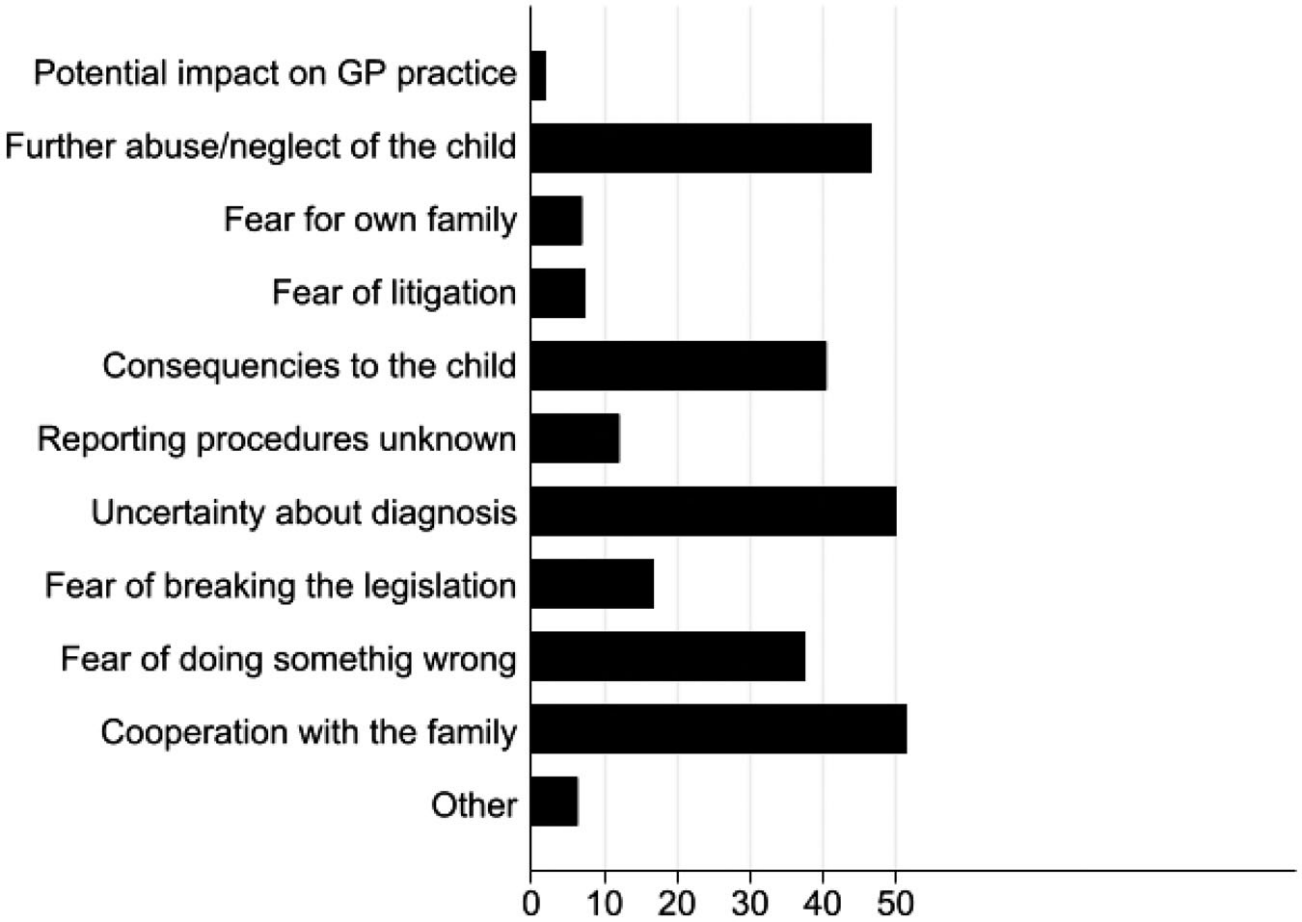
Percentage of responders reporting factors affecting the decision to report to social services. Three factors (*fear of breaking the legislation, fear of doing something wrong, cooperation with the family*) were added to the original questionnaire by the authors.

Figure 2 depicts the percentage of responders reporting each of the factors affecting the GPs’ decision to make a report about child maltreatment to social services. Around half of responders reported: *fear that the child will be further exposed to abuse and/or neglect*, *uncertainty about correct diagnosis*, and *collaboration with family*. Less than a tenth was concerned about *impact to their practice*, *fears of litigation*, or *fears for their own family*.

Replies did not differ across types of practice and geographical regions (data not shown).

**Figure 3.**
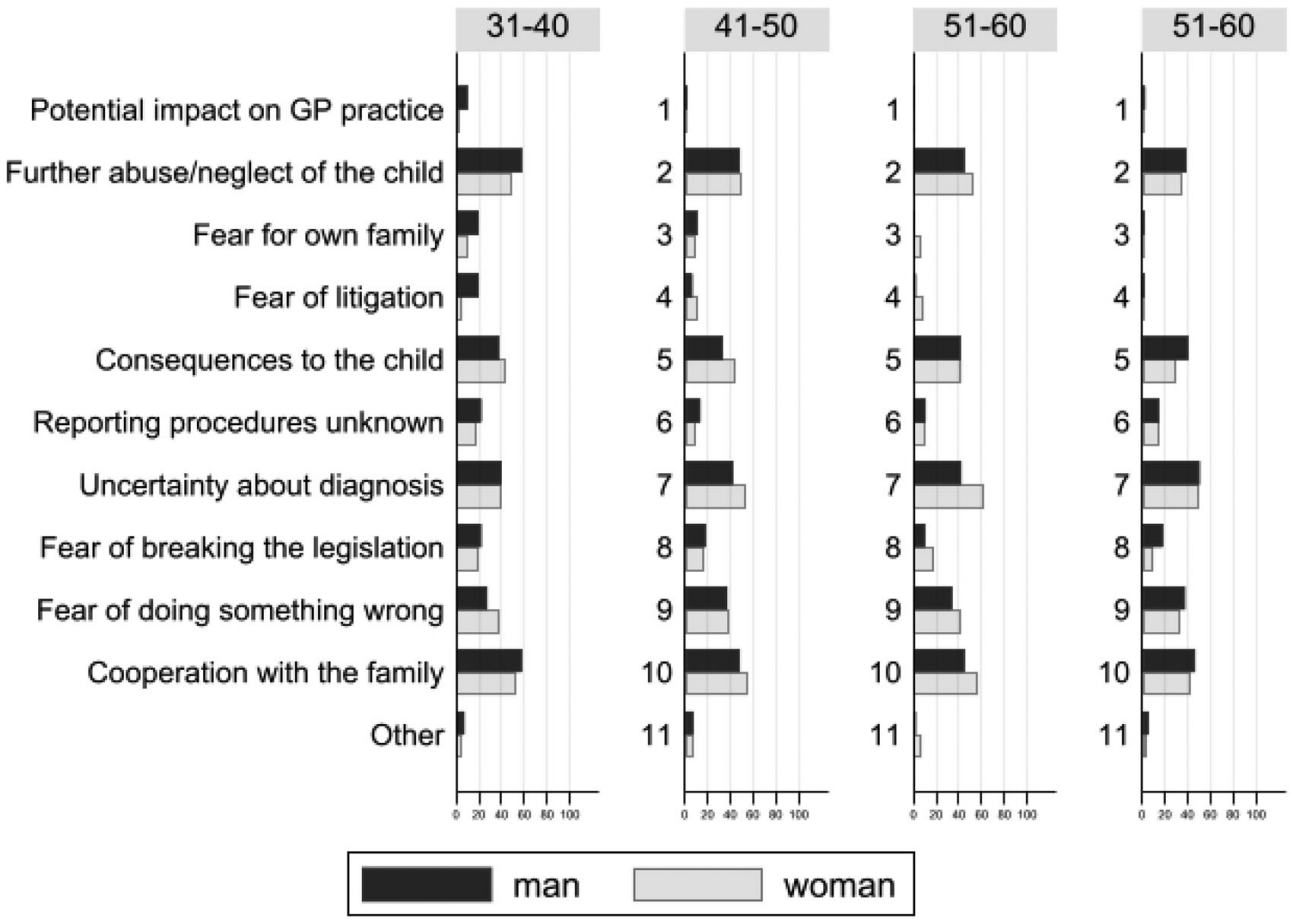
Percentage of responders reporting factors affecting the decision to notify social services according to age and sex of the general practitioner.

Figure 3 depicts the percentage of responders reporting each factor according to age and sex. Some factors showed small sex differences and a pattern of decline with age for both sexes (*potential impact on GP practice, fear for own family, fear of litigation, notification procedures unknown*). However, the overall percentages of GP doctors reporting each factor were roughly of the same magnitude across age and sex strata, and no clear patterns were observed.

#### Interviews and fieldwork – managing suspicions in general practice

Two overarching topics were identified in the qualitative data: rising suspicion (‘something’ not right) and managing suspicion. One key point that stand is that it was often impossible for GPs to figure out what was going on with a child from a single consultation, and one strategy they used was to make follow-up appointments to keep track of the child. This safety net approach was widely used to maintain the relationship with the child and the family that caused some level of concern and to both GPs and PNs one of the greatest challenges was that they feared that losing this trust and connection with the family could potentially harm the child.

I was aware of it even before I had the consultation and thought that it was all really rather strange. So I made a follow-up appointment and said well we just have to follow up on this, I gave some other reason, and I saw him a few times after that, and I still thought something was off, but I didn’t think that there was an obvious reason…. I also asked another GP to take a look, just to take a look and see if he noticed anything. But we didn’t think that there was anything that we could base a report on. (GP 9)

In addition to safety netting, as noted in the above quote, most GPs preferred discussing their suspicions with a colleague before making a report, when they were unsure about what they had observed/sensed, or when they experienced patient cases where they did not know how to act. In these cases, practice nurses would often call their GP into the consultation to observe.

I won’t say that we always do it but in those more difficult cases, I think we need to discuss it, in order to be able to deal with it ourselves. Because when a suspicion is raised it is nice to just get a feeling that it is not just me being paranoid. So, we typically discuss it over lunch, or we knock on each other’s door if we need another set of eyes. (PN 5)

When the concern was particularly vague or if it gave rise to increased uncertainty, the colleague called upon was often a hospital specialist in the paediatric department.

Well, sometimes it can be the way that parents explain the symptoms.… that they are overly concerned or not concerned or when something appears unusual in the interaction… I don’t know if you necessarily think abuse, but one thought could be whether this child is cared for properly. Are the parents able to provide support when they are suffering from whatever… are in pain and so on. And that can also be a reason to refer to the pediatric department where they are able to get that support, right, if they need it. (GP 11)

Thus, referring to the specialists was used as a means of support, and rather than reporting GPs would often refer, when they had the feeling that something was wrong with the child or a family, but they were unsure about how to pinpoint their uncertainty.

Sometimes I chose to refer because I am really uncomfortable with the situation. And at the paediatric department they will be like, well there is nothing here…. No but we do have to observe the situation for more than the 10 minutes we have here in general practice. (GP 2)

One finding that featured throughout the interviews was how GPs and PNs were centrally placed in terms of being the patients’ health and care coordinator [*tovholder*]. If the families had nowhere else to go, they would seek help from their GP.

Just the other day one of my patients came in, a man, and he had just been contacted by the social services, because someone had reported that they thought that he was not caring properly for his children. And he came to me to ask what to do in that situation right. In fact, we are kind of society’s dust bin, right, if no one else will help then your GP will. (GP 7)

GPs and PNs valued this cooperation with patients and families and referred to themselves as one of the most consistent figures in the lives of vulnerable families. Not losing touch with those families was important to them.

I think that we are very central because we are so stable…. In fact, we are more stable than the people from the municipality right. They know us and we know the families – for different things. Not only because of the child but we know the father for his issues and the mother for hers. And the child well visits are also a way of forming a bond. So we are considered more as on their side. (PN 1)

In most of the interviews particularly the GPs pointed out that there are challenges inherent in the interaction between general practice and social services in the municipality, which is the unit responsible for managing reports of suspicions of child maltreatment. One barrier was communication across the sectors. GPs were often unaware of the actions taken by social services after a report was made.

There are several challenges with cooperation on child care ……. For instance that we get no response on our reports…. well now at least they have started sending an acknowledgement of receiving the report. (GP 4)

Most GPs found cooperation with social services difficult, and the lack of response was frustrating. The GPs did not consult with social services when in doubt, and primarily reported on cases where there were concrete observations, and in most cases, reports were made in cooperation with the family, as a way of getting help to a family in need.

## Discussion

We combined questionnaire and ethnographic data and explored the ways suspicions of child maltreatment are managed in Danish general practice. Our results show that most GPs (94.2%) prefer to report to social services in cases of suspicion of child abuse and/or neglect. However, before making the report many GPs prefer to discuss the case with a colleague, especially GPs who were younger, female, and working in group practices. The PNs never made the referral on their own, this was always done by the GP. However, the management of suspicions of child maltreatment were similar across the professions. Generally, the findings from the questionnaires in our study were similar across type of practice and Region. The qualitative data supported and expanded these findings, highlighting the challenges of communication with social services and the very limited opportunities for collaboration around the child and family. The questionnaires showed that only a few GPs (8.8.%) preferred discussing cases with social services before making a report, possibly because of a lack of response or feedback. The ethnographic data provided an in-depth understanding of the feelings of uncertainty expressed by GPs and PNs, especially when their concerns were based on a feeling that something was off, and not on clear-cut signs. In these cases they did not seek advice or support from social services, but when they are in doubt about their findings, they preferred to refer to the paediatric department or discuss their concern with colleagues, while trying to maintain their relationship with the family. The GPs and PNs stressed that they were often one of the few professionals who had a longstanding relationship with the families who struggled the most, and making new appointments with the child or family were used actively as a strategy to keep track of the child.

## Strengths and limitations

The two different methodologies uniquely supported each other. The ethnographic data provided in-depth perspectives on the findings of the questionnaire responses and elucidated the difficult processes around reporting to social authorities from a GP setting. These perspectives seemed supported by the responses of most participants to the questionnaire.

The questionnaire response rate was, as seen in similar studies, quite low, thus raising questions of representativeness and generalisability of the results. Although the responders were similar to the background population, some underrepresentation of GPs over 60 years of age and GPs working in the Capital Region occurred. We have no data to evaluate whether the responders differed according to their attitudes and experiences in dealing with child maltreatment compared to the non-responders and can thus not preclude selection bias with respect to this. The participants in the ethnographic study were selected based on practice type, patient population and geographic location, which may have reduced the potential selection bias.

Combining the two data sources should of course be considered with caution, as the interviews and observations should not be read as a validation of the questionnaire responses, nor vice versa. The ethnographic data do, however, provide context and depth to the overall patterns that can be observed in the questionnaires, and our results should be interpreted from this perspective.

## Comparison with existing literature

According to both questionnaires and interviews, on most occasions the GPs discussed their concerns and their intention to make a report with the child’s caregivers and made the report in collaboration with the family, a finding reflected in another recent study [14]. Although this transparency seems positive, and points to the negotiations around patients’ life circumstances, specific situations and contexts, GPs may still be unable to follow up on the family after making a report. Without feedback or support, other than referring the family to social services or the hospital paediatric departments, and without established channels to consult with other professionals on their concerns, a core concept expressed by the GPs in our study was the feeling of uncertainty. This feeling may be further enhanced by relatively limited experience with cases of abuse and neglect in general practice, suggesting that other doctors may be the most important network GPs somewhat haphazardly use to deal with difficult cases. The questionnaire did not differentiate which colleagues, from hospital or practice, the GPs prefer to discuss cases with, but in the interviews, it was obvious that many did rely largely on colleagues from their own practice, albeit specialist departments at the hospital were used as a safety net. The GPs used both telephone advice and referral to the specialist departments as second opinions, rather than referring to the social services, which may further delay the assistance to a child in need of help. Interestingly, GPs working in single practices did not consult colleagues to the same extent as GPs from group practices, which may of course reflect the significance of availability when GPs involve colleagues. Nevertheless, it may also indicate that they consult less frequently with the specialist departments, which may be considered an example of how reasonable suspicion means different things to different people, as suggested by the authors of a US study [27]. They show that young females with fewer reports during the past 2 years had a more substantive and conceptual understanding of reasonable suspicion. In the pilot study we carried out prior to this study, we also found the significance of experience reflected in tendencies to report and refer on suspicions [14].

Contrary to other studies [13], considerations and fear for personal or professional impact seem to have little influence on GPs decisions to report. This may reflect both willingness to run risks while caring for patients and feeling safe to make difficult choices when necessary. The key factors affecting GPs in their decision to make a report were either centred around the child and the family or related to uncertainty. They included fear of triggering an unstable family and thereby causing further harm to the child, fear for future collaboration with the family, fear that the child could be worse off if social services intervened or were related to an inborn uncertainty about the diagnosis. Many of these factors are hard to cover in guidelines, which are the most often suggested tools to assist clinicians in situations of uncertainty. As pointed out by Stolper et al. [15,p.122], while GPs are often blamed for low reporting rates for child maltreatment, this does not mean that the detection rate is low. Insights from social sciences have pointed out how ‘*control and uncertainty are always negotiated within social relations’* [24,p.11], which may be related with how GPs try to improve the child’s situation by making use of the doctor-patient relationship and by involving other professionals, such as paediatric departments. Our results indicate that it is not necessarily that GPs and PNs do not discover or suspect that things are not right. They manage their uncertainty by referring to specialists, by working on relations with the family, or by watchful waiting. However, if the GPs are to act early this uncertainty must be acknowledged and perhaps incorporated into guidelines and teaching curriculum for medical students and GP trainees, in order to enable proactive attention to child maltreatment.

## Implications for practice

If uncertainty is taken seriously as a central and intrinsic aspect of acting on suspicions of child maltreatment, we may be able to better assist GPs in acting early and proactively in those situations where there are no cuts or bruises, but still ‘something’ which alerts their attention. Moreover, it seems important to establish a better relation with, and understanding of, the responsibilities of social services, what reporting to them means, and how this might help the child and the family.

## Conclusion

There is little doubt that the complex reality of general practice provides an important but also difficult point of departure for detecting child maltreatment. It seems vital to improve the communication, transparency, collaboration, and feedback between general practice and social services in order to improve child welfare. GPs and PNs often feel left to themselves in managing their suspicion and do not consult with social services when in doubt, although social services are the responsible authorities for children at risk of maltreatment. Reacting to the suspicion of child maltreatment in general practice holds the potential of caring for children who are subjected to neglect and/or abuse much earlier than when these children are seen by doctors at the more specialised departments, who rarely meet the child until the impairment is severe.
